# Complete Genome Sequence of *Lactobacillus rhamnosus* Strain BPL5 (CECT 8800), a Probiotic for Treatment of Bacterial Vaginosis

**DOI:** 10.1128/genomeA.00292-16

**Published:** 2016-04-21

**Authors:** Empar Chenoll, Francisco M. Codoñer, Juan F. Martinez-Blanch, Daniel Ramón, Salvador Genovés, Marco Menabrito

**Affiliations:** a Department of Food Biotechnology, Biópolis SL, Parc Científic Universitat de València, Paterna, Valencia, Spain; b Lifesequencing SL, Parc Científic Universitat de València, Paterna, Valencia, Spain; c Ferring International Center S.A., Saint-Prex, Switzerland

## Abstract

*Lactobacillus rhamnosus* BPL5 (CECT 8800), is a probiotic strain suitable for the treatment of bacterial vaginosis. Here, we report its complete genome sequence deciphered by PacBio single-molecule real-time (SMRT) technology. Analysis of the sequence may provide insight into its functional activity.

## GENOME ANNOUNCEMENT

Bacterial vaginosis (BV) is one of the most common disorders in women of reproductive age, and can be microbiologically characterized by dysbiosis, where the lactobacilli-predominant vaginal microbiota is overwhelmed by an overgrowth of pathogenic organisms (1).

In this case, the strain *Lactobacillus rhamnosus* BPL5 (CECT 8800), isolated from the vaginas of healthy women, shows functional properties in the treatment of vaginal and urogenital tract infections caused by lactobacilli deficiency. Here, we present the complete genome sequence of this probiotic strain.

In order to carry out the complete genome sequencing of the strain *L. rhamnosus* CECT 8800, massive sequencing technology was implemented using the PacBio platform (Pacific Biosciences, Menlo Park, CA). A 10-Kb library was constructed with purified DNA, and one single-molecule real-time (SMRT) cell was sequenced using XL-C2 chemistry with a data collection time of 120 min. The sequencing run provided a total of 150,292 sequences with an accuracy of Q20. Sequences obtained were filtered by quality and a total of 51,060 sequences were obtained, with a mean read length of 13,408 nucleotides (nt) of Q20 quality. The total data output was 684 Mb. *De novo* assembly employed the default parameters of the Hierarchical Genome Assembly Process approach (HGAP). Reads numbering 4,901 of around 7.8 Kb were obtained, providing a single contig of 3,024,027 base pairs and coverage of 178×. No plasmids were detected.

The assembled genome sequences were annotated using the Prokka annotation pipeline, version 1.11 (2), which predicted tRNA, rRNA, and mRNA genes and signal peptides in the sequences using Aragorn, RNAmmer, Prodigal, and SignalP, respectively (3–6). Putative gene products were then assigned to the protein-coding genes (CDSs) based on their similarity to sequences in the respective databases. The predicted CDSs were further annotated by BLAST searching against the MvirDB database of virulence factors and the CARD database of antibiotic resistance genes. Associated gene ontology (GO) terms were obtained by matching them to the reference proteins in the Swiss-Prot database.

The genome contains 2,890 elements, of which 2,814 are open reading frames (ORFs) (2,292 canonical and 522 noncanonical) and 76 are structural RNAs (sRNAs) (15 rRNA and 61 tRNA). The *L. rhamnosus* CECT 8800 genome was compared with the commercial strain *L. rhamnosus* GG genome (ATTCC 53103 [7], genome accession number AP011548.1) with BLASTp (8). In this comparison we detected 392 elements missing in probiotic strain *L. rhamnosus* GG. The analysis of the complete genome of *L. rhamnosus* CECT 8800 may help us to understand the mechanisms involved in its effect against bacterial vaginosis.

### Nucleotide sequence accession numbers.

The results of this whole-genome project have been deposited at DDBJ/EMBL/GenBank under the accession no. LT220504. The version described here is the first version.
